# Experiences of a Community-Based Digital Intervention Among Older People Living in a Low-Income Neighborhood: Qualitative Study

**DOI:** 10.2196/52292

**Published:** 2024-04-25

**Authors:** Si Yinn Lu, Sungwon Yoon, Wan Qi Yee, Nerice Heng Wen Ngiam, Kennedy Yao Yi Ng, Lian Leng Low

**Affiliations:** 1 Dalla Lana School of Public Health, University of Toronto Toronto, ON Canada; 2 Health Services and Systems Research, Duke-NUS Medical School Singapore Singapore; 3 Center for Population Health Research and Implementation, SingHealth, Regional Health System Singapore Singapore; 4 Population Health and Integrated Care Office, Singapore General Hospital Singapore Singapore; 5 Department of Internal Medicine, Singapore General Hospital Singapore Singapore; 6 TriGen Ltd Singapore Singapore; 7 Division of Medical Oncology, National Cancer Centre Singapore Singapore Singapore; 8 SingHealth Duke-NUS Family Medicine Academic Clinical Program, Duke-NUS Medical School Singapore Singapore

**Keywords:** digital divide, digital learning, smartphones, social gerontology, older adults, COVID-19 pandemic, technology adoption

## Abstract

**Background:**

Older adults worldwide experienced heightened risks of depression, anxiety, loneliness, and poor mental well-being during the COVID-19 pandemic. During this period, digital technology emerged as a means to mitigate social isolation and enhance social connectedness among older adults. However, older adults’ behaviors and attitudes toward the adoption and use of digital technology are heterogeneous and shaped by factors such as age, income, and education. Few empirical studies have examined how older adults experiencing social and economic disadvantages perceive the learning of digital tools.

**Objective:**

This study aims to examine the motivations, experiences, and perceptions toward a community-based digital intervention among older adults residing in public rental flats in a low-income neighborhood. Specifically, we explored how their attitudes and behaviors toward learning the use of smartphones are shaped by their experiences related to age and socioeconomic challenges.

**Methods:**

This study adopted a qualitative methodology. Between December 2020 and March 2021, we conducted semistructured in-depth interviews with 19 participants aged ≥60 years who had completed the community-based digital intervention. We asked participants questions about the challenges encountered amid the pandemic, their perceived benefits of and difficulties with smartphone use, and their experiences with participating in the intervention. All interviews were audio recorded and analyzed using a reflexive thematic approach.

**Results:**

Although older learners stated varying levels of motivation to learn, most expressed ambivalence about the perceived utility and relevance of the smartphone to their current needs and priorities. While participants valued the social interaction with volunteers and the personalized learning model of the digital intervention, they also articulated barriers such as age-related cognitive and physical limitations and language and illiteracy that hindered their sustained use of these digital devices. Most importantly, the internalization of ageist stereotypes of being *less worthy* learners and the perception of smartphone use as being in the realm of the privileged *other* further reduced self-efficacy and interest in learning.

**Conclusions:**

To improve learning and sustained use of smartphones for older adults with low income, it is essential to explore avenues that render digital tools pertinent to their daily lives, such as creating opportunities for social connections and relationship building. Future studies should investigate the relationships between older adults’ social, economic, and health marginality and their ability to access digital technologies. We recommend that the design and implementation of digital interventions should prioritize catering to the needs and preferences of various segments of older adults, while working to bridge rather than perpetuate the digital divide.

## Introduction

Globally, the COVID-19 pandemic has engendered unprecedented challenges for older adults. In addition to confronting the risks of infections and death [1], prolonged social distancing measures have worsened the physical and mental health as well as the quality of life in the older adult population. During this period, heightened loneliness, social isolation, and anxieties were attributed to reduced social engagement and support, mistreatment, and misinformation [2-4]. Loneliness is associated with higher risks of depression, functional and cognitive decline, and all-cause mortality and morbidity [5-7]. Older adults with lower socioeconomic status, preexisting comorbidities, and poorer access to health care were more likely to experience mental health–related problems [8,9]. Digital technology has surfaced as an essential way for older adults to cope with restrictions and mitigate loneliness by fostering social connections [10,11]. Studies have demonstrated the potential for technology to reduce perceived isolation via improved social support, connectedness, and engagement in activities [12,13] as well as promote a sense of autonomy and confidence [14]. Older adults who had more frequent digital contact, for example, through video calls, social media, or phone calls with friends and family, during the pandemic reported higher levels of satisfaction and well-being and a lower sense of social isolation [7,15,16]. Digital interventions have also been developed to enhance the psychosocial well-being of older adults. For example, a digital human facilitator was found to be feasible and acceptable in alleviating older adults’ stress and loneliness [17], and a digitally guided group intervention increased their digital literacy and abilities to cope with distress [18].

However, a large proportion of older adults globally continue to have little or no access to digital technology [19,20]. With the accelerated digitization of basic services (eg, financial transactions, health, and communications), the growing digital divide may further worsen the inequities in health and exclude the already marginalized segments of the population. Inequalities in access to digital literacy exist not only based on age but also based on income, education, sex, disability status, and immigration status, which culminate in “distinct positionalities of privilege and disadvantage,” shaping diverse attitudes and behaviors toward digital technology use [21-23]. Those with higher incomes across all age groups tend to adopt communication technologies earlier and more extensively than those with lower incomes [22]. In Singapore, older adults with poor health, with less education, and residing in low-income housing were found to experience greater difficulties with internet use [24].

The unequal access to digital technology and challenges associated with its adoption among older adults have been well established in the literature [25,26]. Studies have also reported factors, both at the individual and environmental level, that facilitate or hinder older adults’ adoption of and continued engagement in digital technology [27]. While the factors influencing digital technology adoption among the older adult population have been well documented, there remains a gap in understanding the diverse attitudes, experiences, and perspectives among distinct subgroups of older adults [27]. In addition, much of the discourse and theoretical foundations regarding technology use was informed by conventional models of technology adoption (eg, Technology Acceptance Model and Unified Theory of Acceptance and Use of Technology) or behavioral health theories (eg, Theory of Planned Behavior and Social Cognitive Theory) [28]. While these models illuminate the influence of psychosocial and behavioral factors on technology adoption, they fail to account for the socioeconomic and environmental contexts that shape these attitudes and perceptions toward digital technology.

To fill this gap, this study aimed to explore the experience of and perceptions toward a community-based digital intervention among older adults living in low-income neighborhoods during the COVID-19 pandemic. Particularly, we sought to understand the motivations, perceived challenges regarding the adoption and continued use of smartphones, and benefits derived from the learning process, all in the context of social and economic vulnerabilities. The findings will inform how older adults who are considered vulnerable can most benefit from digital technology adoption and how program implementers can tailor the design of the interventions to older adults’ needs to maximize their effect.

## Methods

### Overview

This qualitative study was part of a larger mixed methods study conducted to evaluate the impact of a community-based digital intervention, namely, Project Wire Up, on older adults’ digital literacy and health-related outcomes in low-income neighborhoods in Singapore. This study used a generic qualitative methodology because the aim was to explore how older adults interpret and attribute meaning to the community-based digital intervention and smartphones [29]. Aligned with a constructivist epistemology, this methodology was selected to emphasize and foreground older adults’ subjectivities and their experiences regarding the intervention.

### Ethical Considerations

Ethics approval to conduct the study was obtained from the SingHealth Centralized Institutional Review Board (2020/2722). Ethical considerations were accounted for in the design and implementation of the study within the context of a marginalized older adult community. For example, as in-depth interviews with older adults regarding the challenges they face in their everyday lives may elicit sensitive or upsetting recalls, interviewers will provide options for receiving various forms of support when needed (eg, taking breaks, postponing the interview, and options for referral to mental health resources) and remind participants that responding to interview questions is voluntary. In addition, as participants are referred to the study upon completion of the digital intervention, they may feel obliged to enroll in the study. Thus, the study team will highlight that participation in the study is voluntary and will not influence their access to services or ability to participate in subsequent community interventions. Informed consent was obtained before the interviews.

### Setting

Singapore is experiencing an accelerated rate of population aging [30]. By 2030, the country will have one of Asia’s oldest populations, with one-fourth of the population aged >65 years. During the COVID-19 pandemic, heightened safety measures were implemented from April 2020 to June 2020 to curb widespread transmission. Nonessential services were stopped, and movement in public spaces was significantly curtailed. As most of the populace turned to digital means to maintain social connections and keep up to date with the news, a digital divide based on age became palpable [31]. Concerns that older adults would be *left behind* in the digital world became an impetus for a slew of government programs such as the Seniors Go Digital [32] to encourage smartphone adoption among this group [33,34]. Considering the heightened digital exclusion experienced by older adults with socioeconomic disadvantages, efforts were dedicated to enhancing access to digital literacy for this group of older adults [35].

Project Wire Up was established in July 2020 by TriGen, a voluntary organization, in partnership with Singapore General Hospital, Infocomm Media Development Authority, and older adult activity centers in Singapore to pilot a volunteer-led, one-on-one, home-based digital literacy program [35]. Those enrolled in Project Wire Up were eligible to purchase smartphones at subsidized rates and were matched to volunteers who provided one-to-one coaching regarding digital skills, including making video calls, connecting to Wi-Fi, performing web-based purchases, and using government services. An average of 6 sessions were conducted at participants’ homes over 3 months and were personalized according to their interests and competencies. Upon the completion of the program, volunteers may connect older adults to formal and informal networks for social support [35]. The aim of the intervention was to close the digital gap and improve social connectivity among older adults with lower socioeconomic status living in public rental neighborhoods. Public rental housing units, a sensitive indicator of area-level socioeconomic status in Singapore, are heavily subsidized flats that cater to lower-income households. Individuals eligible for public rental housing have total household gross monthly incomes that do not exceed approximately US $1000 [36].

Older residents in public rental flats face higher risks of frequent hospital admission and readmission, higher use of hospital and emergency department services, and longer durations of hospital stay [37]. Living in rental flats has also been correlated with poorer physical and mental health, including poorer cognitive function and higher depression rates among older adults [38]. In addition to shouldering a higher disease burden, they are more likely to have limited social and financial support. As poorer digital literacy may negatively influence older adults’ health via weaker social connections [39] and impact their ability to access health information and increasingly digitized modes of health care delivery [20], Project Wire Up’s primary goal of enhancing social connectivity and digital literacy has the potential to improve older adults’ well-being and mitigate health care use.

### Recruitment and Data Collection

The study team worked closely with the implementation team to recruit eligible participants who met the inclusion criteria of being aged ≥60 years and successful completion of the program. Participants aged ≥60 years or who had not completed the intervention were excluded. A list of eligible participants was referred to the study team, who then contacted the participants to ask whether they were interested in participating in the study. A purposive sampling technique was used to obtain a diverse sample of participants, in terms of ethnicity, sex, and language spoken, to reflect the heterogeneous older adult population in Singapore. Between December 2020 and March 2021, the study team conducted semistructured interviews at various public rental housing estates. During the interview, participants were asked about their life histories, daily routines, challenges they faced during the lockdown, support received, meanings associated with smartphone use, and their experiences and challenges in participating in the program. Overall, 2 interviewers trained in qualitative research conducted the interviews, while observing the appropriate social distancing measures. Interviews usually lasted between 45 minutes and 1.5 hours and were conducted in English or local dialects (Mandarin, Cantonese, Hokkien, and Malay) and audio recorded.

### Data Analysis

All interviews were transcribed and translated from local dialects to English. Then, the transcripts were coded using NVivo (version 12; Lumivero). Consistent with a generic qualitative methodology, a thematic analysis was conducted. An initial codebook was generated based on key sections of the interview guide. The interview guide drew on certain concepts from the existing literature, which posit that factors such as health, social network, and perceptions about technology influence how participants relate to and perceive smartphone use. These initial codes also included the challenges faced during the COVID-19 pandemic, as we anticipated that the pandemic may influence older adults’ attitudes and behavior toward technology. Participants were also asked about their experiences with the program, including preprogram expectations, memorable moments, preferred mode of learning, postprogram smartphone use, and what they hoped to learn in future. These program-specific questions were intended to inform the design and implementation of future interventions.

Using this initial codebook, 2 coders coded 3 transcripts together through inductive and inductive coding methods to identify regularities in ideas and other emergent themes that may be relevant to the research problem. In this process, existing categories were expanded, and new categories were added to the codebook. Then, both coders coded the transcripts separately using the reviewed codebook. As both coders have each conducted several interviews with study participants, they had an in-depth understanding of and familiarity with the data and conducted regular discussions after coding 4 to 5 transcripts to resolve any discrepancies and discuss whether the key analytic categories that emerged were reflective of the meanings expressed by the participants. Analytic memos were also written alongside the coding process to reflect on any issues that arose during the coding process, emergent patterns, and thematic categories and subcategories [40]. Constant comparative analysis was performed throughout the process to compare the interview data to emerging categories and to determine the consistency in coding. Categories were created and refined when the data did not fit into categories. Then, the coders, together with members of the study team, discussed the key themes that were most salient in the interview data that shaped older adults’ experiences with the intervention and smartphone use. This paper followed the COREQ (Consolidated Criteria for Reporting Qualitative Research) checklist to ensure comprehensive and transparent reporting of the results [41].

## Results

### Participant Characteristics

We conducted a total of 19 interviews over a period of 4 months. Data saturation was achieved at 15 interviews, and we conducted a few more interviews to ensure that new data did not disclose new insights. Of the 19 participants, 12 (63%) were female participants, 16 (84%) were Chinese, 13 (68%) were widowed or single, and 15 (79%) lived alone in 1-room rental flats. Most participants reported a relatively low educational attainment, where 32% (6/19) of the participants received no formal education, and 26% (5/19) received primary education. The participants’ characteristics are presented in Table 1.

In this study, we identified three themes that reflect the perceived challenges and benefits related to learning the use of smartphones among older adults who live with low income: (1) age and social marginality, (2) technological design as a form of exclusion, and (3) digital learning process as a tool for mitigation of social isolation and marginality.

**Table 1:** Participants’ characteristics (N=19).

Characteristics		Participants, n (%)
**Age (years)**		
	60-69	5 (26)
	70-79	7 (37)
	≥80	7 (37)
**Sex**		
	Male	7 (37)
	Female	12 (63)
**Ethnicity**		
	Chinese	16 (84)
	Malay	1 (5)
	Indian	2 (11)
**Marital status**		
	Married	4 (21)
	Widowed	6 (32)
	Divorced	2 (11)
	Never married or single	7 (37)
**Highest level of education**		
	No formal education	6 (32)
	Primary	5 (26)
	Secondary	5 (26)
	Vocational or diploma	1 (5)
	University and above	1 (5)
	No response	1 (5)
**Housing type**		
	1 room	15 (79)
	2 rooms	3 (16)
	3 or 4 rooms	1 (5)
**Living arrangement**		
	Staying with spouse only	3 (16)
	Staying alone	15 (79)
	Staying with helper	1 (5)
**Employment status**		
	Working (full time)	2 (11)
	Retired	13 (68)
	Unemployed	4 (21)

### Age and Social Marginality

#### Overview

Older adults’ perceptions about smartphones and the digital intervention must be understood within the context of their experiences with aging and social precarities. Precarity refers to an existence characterized by insecurity, unpredictability, and vulnerability that could emerge at an intersection of social disadvantages that extend into later life [42]. This means that older adults’ everyday experiences of aging must be understood and situated within the social structures that they are embedded in. In this study, most participants were in the middle-old category and experienced social and income-related vulnerabilities. Thus, our analysis demonstrated that the manner in which older adults relate to smartphone use and define their capacity as learners is inextricably connected to their age-related anxieties and perceived socioeconomic positioning.

#### Aging-Related Precarities and Internalized Ageism

The most cited barrier to learning was age-related cognitive and physical decline, including visual and hearing impairment, memory difficulties, and decreased agility in their fingers, which impeded the ability to use the smartphone and retain knowledge from each session. More than half of the participants felt embarrassed about not being able to remember what was taught. For example, some participants described the anxieties and feelings of helplessness related to a perceived “deterioration” in their minds that made it difficult to absorb and apply the information learnt—a problem they attributed to old age:

You are young, your minds are good, you can put many things inside but our mind is deteriorating, old already. [I] cannot see, cannot walk fast, this is natural for every person who gets old.

Participant #3, female

In addition to the challenges related to cognitive and physical constraints, participants also expressed negative aging self-perceptions throughout the interview. While these were not explicitly stated as barriers to smartphone learning, they reflect the attitudes toward learning or negative associations between learning and old age. For example, a participant expressed the futility and meaninglessness of learning during old age:

At first, I did not want to take [the phone], [but] the manager at the SAC said, “take it, learn slowly.” I said I am already going to die, no point learning, so old already, no one will know what will happen tomorrow, just live one day at a time.

Participant #6, female

Our participants considered their unsuccessful attempts at mastering smartphone use as a sign of their inaptitude due to old age and the futility of the learning process. Participants had a strong tendency to individualize responsibility for the outcome of smartphone learning. Despite feeling uncertain about their ability to sustain smartphone use, participants chose not to seek help from volunteers after each visit due to their fear of “troubling” them and their reluctance to be seen as a “burden.” For example, some participants assessed themselves as learners who were “not worthy” of the time and attention from the volunteers who have “better things to do”:

I said as a volunteer, you need to work, if you come here it takes about 1 hour to teach us, we are wasting your time, how much can you teach us, after you leave, I cannot remember already, now we are old...I do not want to obstruct people’s time...We do not want to trouble others.

Participant #1, male

Therefore, participants internalized ageist assumptions by viewing age-related limitations as “deficits” that prevent them from undertaking smartphone-related activities [43]. Such ageist self-judgments exacerbate their poor self-conception, low self-worth (ie, as learners with no scope for growth and whose needs should not be prioritized), and resistance toward learning.

#### Social Precarities and Self-Imposed Stigma

Participants’ perceived lack of interest and confidence to learn smartphone use were also shaped by the awareness of their social positioning in relation to other older adults. Overall, one-third of the older adults emphasized that it was particularly difficult for them to learn to use the smartphone because of language barriers and illiteracy. For example, 1 participant was quick to distinguish himself from those who spoke English and were literate—characteristics that he felt predisposed them to increased competency and ability to acquire smartphone skills at a faster pace:

Those [literate] people know words, know English, know the language. It is different, teaching them is very fast. For us, we do not recognize words, you teach me 10 times but I cannot remember...Waste time, waste effort.

Participant #1, male

Most Chinese older adults residing in the neighborhood where the intervention was conducted are Mandarin-speaking or dialect-speaking individuals, making it challenging for them to navigate the smartphone. While volunteers helped these older adults change the default language setting to Mandarin, some participants were still not able to identify the characters due to their limited literacy. Participants viewed smartphone use as being in the realm of the privileged “other,” which does not align with their identities as “low income” or “uneducated.” For example, when asked how she feels about using the smartphone for purposes such as seeing the physician will change her life, 1 participant expressed that smartphones were not suitable for the “kind of people” living in her neighborhood:

Some of us are uneducated, if educated, they have means or help and the ability to have a higher [paying] job. They will not be living in this area, you must understand what kind of area people live and what kind of people are living inside here, it’s not only when you think it’s good you can see on TV it’s good for old people.

Participant #13, female

By perceiving that smartphone use is not applicable to older adults living in a certain “kind of area,” some participants “classified” themselves as failing to belong to the “in group” of digitally savvy older adults, thus reducing their self-esteem and motivation to learn.

### Technological Design as a Form of Exclusion

Participants also expressed challenges related to smartphone technology, with its design primarily catering to the needs of a certain type of digitally literate individuals. Many participants described their interaction with the device as a stress-inducing process, pointing out that the sensitivity of the touch screen, small font sizes, multiple apps, and colorful esthetics made it confusing to navigate the interface. For example, 1 participant lamented that a combination of poor eyesight and stiff fingers resulted in her accidentally dialing the wrong numbers, incurring the wrath of family and friends:

The smartphone is very sensitive, my finger accidentally touches it, then the other person’s phone will get it, so that is the trouble for [an] old lady...[my] eyesight is very poor, if our eyesight is poor and the writing is so small, how you expect us to see...This phone is sensitive. That’s why I told you it’s not suitable for us old people.

Participant #13, female

Thus, participants noted the incompatibility of smartphone’s functions with the needs of older adults and suggested that smartphone use will be helpful for those in the young-old group but felt that learning at an older age may not be useful. The failure of some smartphone designs to consider older adults’ age-related cognitive and physical limitations, needs, and preferences may also account for their sense of ambivalence toward smartphone use. For example, some participants expressed a disconnect between the prospects of learning a new, potentially disruptive technology and the fundamental priorities that they have at this point in their lives.

When asked what it means to be healthy, dominant themes expressed by participants included the ability to “walk,” ability to eat as they desired, having a “clear” mind and good eyesight, absence of ailments and difficulties, and ability to “live day by day doing the things [they] enjoy.” Therefore, the purportedly transformative potential of smartphones was incongruous with what participants valued or perceived as essential to their current life situation:

I want my life to be as simple as possible, do whatever I want to do...I just want to be happy, my mind has no space for other complicated things. At my age, I do not know when I am leaving this world, learn already also no point.

Participant #8, female

We do not use these phone applications, only the youngsters use, there is a camera, take whose picture? A lot of these games, play for what...? Young people like all these funny things, we old people only need big font, big screen, simple.

Participant #3, female

However, 11% (2/19) of the participants were motivated by the opportunity to learn new skills and expressed comfort and familiarity with navigating the smartphone. Their motivations were primarily shaped by active early-life work experience and the desire to keep in contact with closely connected family networks. Support from family members also facilitated their experience of uptake and sustained use. This aspect holds significance as it shows that despite cumulative disadvantages structured by lack of education, income, and employment opportunities, social support could influence their access, familiarity, and motivation to engage in digital learning.

### Digital Learning Process as a Tool for Mitigation of Social Isolation and Marginality

While older adults in this study encountered challenges influenced by their experiences of aging, social precarities, and technological barriers, some participants expressed benefits related to the strengthening of relational ties with new friends or family members.

Many participants stated that their interactions with volunteers were one of the program’s most memorable moments. They appreciated having “someone to talk to” and liked that the volunteers were friendly, helpful, and approachable and provided personalized attention to addressing their queries. Thus, participants enjoyed the social exchanges with the volunteers, particularly if they could “chat and get along very well”:

I miss him [the volunteer], because when I sit here, I miss him sitting next to me, talking to me, teaching me what to do, [he] is a good person, really good, he’s working, he said no problem auntie you can call me if you have any problems.

Participant #15, female

By learning how to make phone calls, send voice messages, and use mobile phone apps, participants mentioned improved social relationships, such as making new friends through the volunteers, and found it easier to communicate with others using the video call function:

It is more convenient to communicate with people, you can see them. Otherwise at that time, we did not know how to use the function, then it felt like we lost contact. You can only hear the voices but not see the people.

Participant #12, male

Thus, given that most older adults in this study lived alone, designing an intervention that not only emphasized acquiring smartphone skills but also concurrently nurtured social bonds and connections appeared to foster participants’ initial acceptance and adoption of digital technology.

## Discussion

### Principal Findings

This study explored the perceptions and experience regarding a community-based digital intervention among older adults residing in a lower-income neighborhood. We sought to understand older adults’ motivations for learning, challenges to uptake and sustained use, and benefits derived from the digital engagement processes in the context of age-related and social disadvantages.

We found that the meanings older adults associated with learning at old age shaped their motivation and confidence in learning. Participants cited cognitive and physical limitations such as visual or hearing impairment and memory difficulties and a perceived sense of futility and meaninglessness of learning at old age as factors that limited their capacity and interest in digital learning. Many older adults expressed embarrassment and frustration regarding not being able to retain or apply what was taught and viewed these challenges as a *natural* outcome of old age. These negative self-perceptions are emblematic of the internalization of ageist structures and stereotypes that associate “being old” with being not technologically savvy. These findings corroborate studies that have documented how older adults’ identification with the negative connotations related to their age group may deter technology use [44,45]. These ageist stereotypes that depict older adults as “inflexible” or unable to “adapt to new ideas and to the use [of technology]” contribute to older adults’ feelings of low self-efficacy and discomfort and beliefs that efforts to learn will be unproductive or embarrassing [46].

Our study also reveals that these age-based anxieties intersect with older adults’ experiences of other forms of marginalization, including perceived stigma of being less educated and literate than other segments of the older adult population. The finding that older adults encounter language barriers aligns with those of a study in Singapore that described how the fear of information and communication technology among older adults in Singapore may be explained by their “limited command of English,” particularly among those with lower levels of education and socioeconomic status [26]. This study also found that older adults in this group expressed concerns related to the affordability of purchasing digital devices [26]. However, a novelty of our findings is that although the presence of subsidized smartphones facilitated smartphone uptake, older adults’ lingering ambivalence and aversion toward smartphone learning is also shaped by their consciousness and perception that smartphone use and technology adoption belongs to a privileged *other* from which they are excluded, and this influences their interest and motivation to learn. While the literature has recognized the importance of income and education in influencing older adults’ intention to use technology [28], our findings contribute to the existing literature by highlighting that older adults may internalize ageist attitudes and stigma resulting from their positionality within the social and economic structure. These self-perceptions hinder their desire to participate in and sustain smartphone learning. In addition, in the context of Singapore, public messages also showcase digital savviness and active social engagement as a marker of successful and healthy aging [33,47]. As studies have shown that engagement in digital practices corresponds to sociocultural conceptions of aging that is “active, engaged, independent [and] highly productive” [48], we posit that broader norms of successful aging may shape the identities, feelings of disempowerment, and negative self-perceptions of older adults with lower income and, in turn, affect their dispositions and perceptions toward smartphone learning.

In addition, our study demonstrates that older adults’ difficulties in navigating the smartphone’s user interface point to the absence of age-friendly features. These findings confirm those of earlier studies that suboptimal design features of digital devices, such as display screens that are challenging to navigate, small icons, and overall “low levels of graphic design adaptation” that fail to cater to the needs of older adults, may completely inhibit access [49]. However, our study adds to the literature by emphasizing older adults’ perceptions that the design and function of smartphones are incompatible with their existing priorities, needs, and visions of “healthy aging” and their feeling that it would be more relevant to the needs of young or middle-aged individuals or those who are digitally literate. While our study reiterates the need to examine the heterogeneity in technology use within the older adult population [50], we also emphasize that older adults’ perceived irrelevance and lack of usefulness of the smartphone in their everyday lives provoke a broader consideration of how existing digital tools and technologies often cater to the preferences and needs of a certain segment of the population, while excluding others.

Our study also highlighted that for older adults typically living alone in low-income neighborhoods, the community-based digital intervention had significant social meanings, creating new forms of social connection and relationships. In addition to improving digital literacy, most participants valued the interaction with the program volunteers. The importance of relationship building within the scope of digital learning is also underscored by the few participants who had demonstrated a strong motivation to learn. Participants in this group consistently practiced and used the smartphone to stay in contact with family and friends. This pattern is evident in studies that indicate the importance of familial and social support, particularly the efficacy of intergenerational approaches in the learning of digital skills, where older adults learn digital skills more readily from their grandchildren [50,51]. Overall, our findings reinforce how older adults’ aversion to digital technologies are shaped by factors such as age-related barriers and negative self-perceptions and a lack of user-friendly digital devices. However, our findings add to the existing literature by highlighting how individual-level factors are intertwined with and situated within the structural vulnerabilities that older adults confront such as age and income-related stigma and marginality. Thus, this study makes an important contribution to the existing theoretical models of technology use. Models such as the Senior Technology Acceptance Model have considered how technological use may differ in the context of older adults’ age-related physical, psychological, and social circumstances that predict their attitudes and behaviors toward digital technology [52]. The Senior Technology Acceptance Model explains that technology adoption is influenced by factors such as older adults’ self-reported health and cognitive ability, social relationships and life satisfaction, and levels of self-efficacy and anxiety toward gerontechnology [49,52]. However, our study’s findings supplement these frameworks by underscoring how the attitudes toward smartphone use among older adult populations that are considered vulnerable must be contextualized within intersecting age-related and income-related precarities that contribute to internalized ageist attitudes and social stigma among older learners, thereby shaping their self-perceptions, motivation, and identities. Existing models of technology use should consider the systemic ageism or exclusion that particular segments of older adults experience that may hinder technology adoption. The consideration of how older adults’ attitudes and behaviors toward digital technology are impacted by their experiences of other systemic disadvantages urges a shift from placing the onus of digital uptake on older adults toward bolstering the existing technological systems and social supports to improve digital connectedness.

### Recommendations for Future Interventions

Based on our findings, 4 key strategies could be recommended to enhance the teaching methods and learning outcomes of older adults in low-income communities, as described in the following subsections.

#### Understanding the Compatibility of Digital Technology With Older Adults’ Lived Experiences

Implementers should consider whether the digital intervention and device are compatible with participants’ needs, preferences, and social circumstances. To comprehend the factors that influence participants’ motivations and attitudes toward learning a new technology, implementers could conduct a needs assessment to identify participants’ healthy aging goals, daily routines, support network, and interests to plan how the smartphone could be relevant to their priorities. Although the current intervention tailors the program according to participants’ interests and abilities (where tier 1 involves learning “basic” phone functions such as video calls and tier 3 involves more “advanced” features such as web-based purchases or using government services), participants may not be able to derive meaning from learning these functions if they are not useful in their everyday lives. For example, if a participant wishes to increase their social interaction, the volunteer can teach them the video call function so that they can make a call to a family member or friend. Smartphone learning should not be seen as an end but as a means to fulfill objectives that are of importance to older adults.

In addition, smartphones may also not be perceived as a “resource” amidst financially precarious circumstances, where the urgent trade-offs in everyday priorities of living (eg, inability to pay for medical bills and uncertainties of welfare apps) mean that smartphone learning and its uncertain “rewards” cannot be prioritized alongside other competing demands on time and energy. Thus, volunteers and older adults should jointly examine ways in which they identify the perceived utility and relevance of the digital innovation in their current routines and life goals. Moreover, it is crucial to recognize that older adults do not necessarily share commonly held assumptions of smartphone as the indisputably “better,” “more convenient,” and “simpler” option; even if they do, they may also not perceive or understand these terms in the same ways. Thus, program implementers should consult older participants about what they value, the types of meanings they ascribe to the purported benefits of the smartphone, and the types of learning approaches that can best meet these needs. Expanding the discourses and meanings attributed to smartphones by different subgroups, particularly individuals considered marginalized, can promote intervention frameworks centered on equity and social justice, thus refuting “structures and systems designed by and for persons in more advantageous social positions” [21].

#### Strengths-Based Approach to Dismantle Ageist Stereotypes

To address older adults’ negative self-perceptions about aging, digital interventions should take a directive approach to dismantle ageist stereotypes before cultivating more independent forms of learning [53]. Opportunities to contemplate age-related challenges should be built into the learning model, enabling older adults to confront their self-perceptions as being a “less worthy” or “incapable” learner. For example, techniques such as motivational interviewing can be adopted by volunteers to better understand older adults’ motivations to learn or resist smartphone learning. Rather than using a deficits-based approach (eg, what older adults do not know), motivational interviewing [54] seeks to affirm participants’ strengths, wisdom, and values and develops a plan toward change based on their own insights. This approach emphasizes the creation of a nonjudgmental, respectful, and compassionate space, where the older adults’ choice to learn or not learn the use of smartphone is not frowned upon or stigmatized. When older adults feel more empowered to learn, they can begin to explore the possibilities of smartphone use and refute the previously held conceptions that technology use conflicts with their identities (ie, not for “someone like them”). At the same time, the encouragement of older adults to learn should not involve coercion or guilt-tripping those who choose not to participate. Efforts must be made to assure older adults that a lack of participation will not deprive them of any other community services or assistance, to reduce the likelihood of older adults participating out of fear or obligation. Moreover, the responsibility should not be completely placed on older adults to actively engage in and keep up with digital practices, where resistance to learning becomes stigmatized or viewed as a burden or sign of “backwardness;” the consequence would be a subversion of the “discourse of empowerment” that digital technology seeks to promote [48].

#### Strengthening Social Ties Through Technology

Our findings suggest that smartphones can be adopted to fulfill relational purposes. Incorporating the cultivation of relational ties, in the form of family members, peers, or volunteers within a digital learning model, can serve to be a “catalytic intermediary” to motivate the use of digital technology [26] and sustain older adults’ interest in the program. In addition, digital technology can act as a medium through which older adults who live alone or lack a supportive social network can expand their social capital by “forming new social relationships or maintaining existing social ties” [26].

Digital technology can also serve as a medium through which older adults can acquire new skills or habits as part of a learning group. For example, a study has explored the application of gamification techniques to encourage older adults to improve digital skills through interactive games with a partner on a touch screen tablet. These games were designed to improve cognitive and motor skills and facilitate social interaction and were found to be effective in improving the acquisition of digital expertise [55]. Thus, intervention models that incorporate problem-solving activities and collaborative peer learning can create an interactive space that nurtures social connections and diminishes feelings of loneliness among isolated older adults in communities considered socially disadvantaged.

#### Ensuring Program Continuity

We found that the lack of opportunities for continued practice and application reduced older adults’ motivation for sustained use of the smartphone following the intervention. Future interventions can provide options to participants based on their levels of interest, skills, and aptitude; this could include connecting older adults to guided learning groups to practice the skills taught or to specific interest groups (eg, playing mahjong on the web). In addition, volunteers can visit the older adults at a fixed time to resolve technical issues that they may have related to phone use.

### Strengths and Limitations

While the literature has documented the challenges faced by older adults in the realm of digital learning, this is the first study that uses a qualitative approach to examine how older adults residing in low-income neighborhoods experience aging and the social and health-related challenges that facilitate or limit their self-efficacy and interest in digital devices. In a global context, there have also been other types of interventions that focus on improving older adults’ digital literacy. For example, in North America, digital literacy training sessions have been conducted in public libraries and community organizations [45]; a 4-month program of weekly computer classes was organized for African American older adults with low income at an older adult community center to gain familiarity with assessing web-based information and privacy issues [56]; and a 4-week digital literacy program was conducted to equip older adults with knowledge about how to navigate their computer (eg, sending emails) during the COVID-19 pandemic [57]. However, to the best of our knowledge, it appears that no study specifically explored the impact of a home-based digital literacy intervention during the COVID-19 pandemic, particularly among older adults with lower income.

One limitation is that we were unable to analyze the data in terms of understanding how these experiences and perceptions regarding the program might have differed across different sociodemographic characteristics—sex, age profiles (young-old and old-old), and health conditions—which could have provided richer insights into the experiences of these subgroups. The distinct experiences of these subgroups and the types of responses needed to address the challenges they face also warrant further research [21]. While our study only considered the context of Singapore, we believe that these findings regarding the role of age and social and material precarities in shaping technological use and the suggested solutions to bridge the digital divide will be theoretically useful in understanding the experiences and perceptions of digital tools among marginalized populations in other contexts.

Studies should be conducted to develop culturally sensitive approaches that can promote digital devices as a potential resource that is relevant to the needs of deprived communities, for example, in ways that can potentially improve the socioemotional and physical health outcomes of individuals or serve as a coping strategy in a precarious environment. Mixed methods studies using implementation science approaches [45] should also assess the maintenance of digital interventions in low-income communities, understand what is suitable for whom, and devise educational frameworks specific to the teaching of digital skills that can empower older learners.

### Conclusions

The findings illuminate the need for community-based digital interventions to be designed with the particularities of the older adults’ lived environment and experiences in mind and the sensitivity that these digital tools only occupy one facet of participants’ lives, alongside other priorities and needs. Further studies are required to understand how these dimensions can be integrated into the intervention to enhance the smartphone’s perceived relevance and utility, without being an unwelcome disruption. Measures aimed at promoting individual-level adoption of smartphones must also be addressed alongside approaches that tackle structural inequities, ageist structures, and stigma that disadvantages one group of older adults relative to others. Regarding those who choose not to participate in the “digital wave,” the society must be willing to find and support alternative solutions to include these older adults in ways that promote social contact, autonomy, and socioemotional well-being—outcomes that technology purports to achieve—while not perpetuating their exclusion.

## Abbreviations

COREQ: Consolidated Criteria for Reporting Qualitative Research
